# Left Atrial Wall Stress and the Long-Term Outcome of Catheter Ablation of Atrial Fibrillation: An Artificial Intelligence-Based Prediction of Atrial Wall Stress

**DOI:** 10.3389/fphys.2021.686507

**Published:** 2021-07-02

**Authors:** Jae-Hyuk Lee, Oh-Seok Kwon, Jaemin Shim, Jisu Lee, Hee-Jin Han, Hee Tae Yu, Tae-Hoon Kim, Jae-Sun Uhm, Boyoung Joung, Moon-Hyoung Lee, Young-Hoon Kim, Hui-Nam Pak

**Affiliations:** ^1^Department of Cardiology, Yonsei University Health System, Seoul, South Korea; ^2^Department of Cardiology, Korea University Cardiovascular Center, Seoul, South Korea

**Keywords:** atrial wall stress, atrial fibrillation, catheter ablation, artificial intelliegnce, rhythm outcome

## Abstract

Atrial stretch may contribute to the mechanism of atrial fibrillation (AF) recurrence after atrial fibrillation catheter ablation (AFCA). We tested whether the left atrial (LA) wall stress (LAW-stress_[*measured*]_) could be predicted by artificial intelligence (AI) using non-invasive parameters (LAW-stress_[AI]_) and whether rhythm outcome after AFCA could be predicted by LAW-stress_[AI]_ in an independent cohort. Cohort 1 included 2223 patients, and cohort 2 included 658 patients who underwent AFCA. LAW-stress_[*measured*]_ was calculated using the Law of Laplace using LA diameter by echocardiography, peak LA pressure measured during procedure, and LA wall thickness measured by customized software (AMBER) using computed tomography. The highest quartile (Q4) LAW-stress_[*measured*]_ was predicted and validated by AI using non-invasive clinical parameters, including non-paroxysmal type of AF, age, presence of hypertension, diabetes, vascular disease, and heart failure, left ventricular ejection fraction, and the ratio of the peak mitral flow velocity of the early rapid filling to the early diastolic velocity of the mitral annulus (E/Em). We tested the AF/atrial tachycardia recurrence 3 months after the blanking period after AFCA using the LAW-stress_[*measured*]_ and LAW-stress_[AI]_ in cohort 1 and LAW-stress_[AI]_ in cohort 2. LAW-stress_[*measured*]_ was independently associated with non-paroxysmal AF (*p* < 0.001), diabetes (*p* = 0.012), vascular disease (*p* = 0.002), body mass index (*p* < 0.001), E/Em (*p* < 0.001), and mean LA voltage measured by electrogram voltage mapping (*p* < 0.001). The best-performing AI model had acceptable prediction power for predicting Q4-LAW-stress_[*measured*]_ (area under the receiver operating characteristic curve 0.734). During 26.0 (12.0–52.0) months of follow-up, AF recurrence was significantly higher in the Q4-LAW-stress_[*measured*]_ group [log-rank *p* = 0.001, hazard ratio 2.43 (1.21–4.90), *p* = 0.013] and Q4-LAW-stress_[AI]_ group (log-rank *p* = 0.039) in cohort 1. In cohort 2, the Q4-LAW-stress_[AI]_ group consistently showed worse rhythm outcomes (log-rank *p* < 0.001). A higher LAW-stress was associated with poorer rhythm outcomes after AFCA. AI was able to predict this complex but useful prognostic parameter using non-invasive parameters with moderate accuracy.

## Introduction

Atrial fibrillation (AF) is a prevalent arrhythmia that significantly increases morbidity, mortality, and economic burden (Kim et al., 2018). However, current rhythm management approaches still have limited efficacy and have inspired substantial efforts to investigate the mechanism of AF (Nattel, 2002). Among the complex mechanisms of AF, chronic atrial stretch causes atrial dilatation and is thought to contribute toward AF progression and atrial remodeling (Nattel et al., 2008). Although left atrial (LA) size is a widely used parameter that reflects the degree of structural remodeling and prognosis of AF rhythm control, some studies have reported inconsistent results (Marchese et al., 2012; Zhuang et al., 2012). Similar to ventricular wall stress or wall tension, LA wall stress (LAW-stress) is a parameter that reflects the tensile stress and strain of the atrial wall (Augustin et al., 2020). Since LAW-stress reflects not only anatomical but also functional aspects of AF, it is expected to provide useful insights into AF mechanisms. However, this physiologic parameter has not yet become popular because it requires variables that are complex and difficult to obtain, such as LA pressure and LA wall thickness, for the calculation (Wang et al., 2011). Recently, new clinical studies utilizing the predictive power of artificial intelligence (AI) have actively increased in the fields of cardiology and electrophysiology (Krittanawong et al., 2017; Kwon J. M. et al., 2020).

In this study, we hypothesized that the complex and invasive variables required to calculate LAW-stress can be replaced with non-invasive common variables using AI among patients who underwent AF catheter ablation (AFCA). We first evaluated the clinical usefulness of LAW-stress in cohort 1, which included data on LA pressure and LA wall thickness measured by customized software. We then evaluated whether the high LAW-stress group estimated by AI in an independent cohort 2 without LA pressure and LA wall thickness had similar clinical outcomes.

## Materials and Methods

### Study Population

**FIGURE 1:**
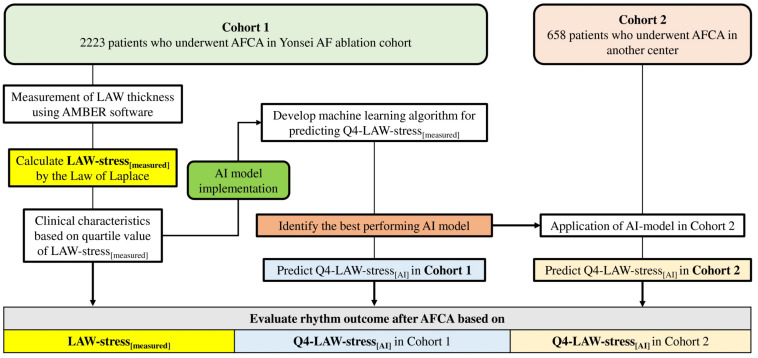
Flow chart of the study analysis. AF, atrial fibrillation; AFCA, atrial fibrillation catheter ablation; AI, artificial intelligence; LAW-stress, left atrial wall stress.

The study protocol adhered to the principles of the Declaration of Helsinki and was approved by the institutional review boards of the Yonsei University Health System and Korea University Cardiovascular Center. All patients provided written informed consent for inclusion in the Yonsei AF Ablation cohort (cohort 1, registered at clinicaltrials.gov as NCT02138695) and the Korea university AF ablation cohort (cohort 2). Cohort 1 included 2223 consecutive patients who underwent *de novo* AFCA. LAW-stress_[*measured*]_ was retrospectively measured in the subjects in cohort 1, and the patients were divided into four groups according to their quartile value of LAW-stress_[*measured*]_. Cohort 2 included 658 patients who underwent *de novo* AFCA (Figure 1). The exclusion criteria were as follows: (1) AF refractory to electrical cardioversion; (2) LA size >55 mm as measured with echocardiography (Verma et al., 2011); (3) AF with rheumatic valvular disease; (4) AFCA using energy sources other than radiofrequency energy; and (5) prior AF ablation or cardiac surgery. All patients stopped all anti-arrhythmic drugs for a period corresponding to at least five half-lives before the catheter ablation.

### Electrophysiological Studies and AFCA

The electrophysiological mapping method and the AFCA technique/strategy used during the study period were consistently performed as described in a previous study (Yu et al., 2017). In brief, an open irrigated-tip catheter [Celsius, ThermoCool SF (Johnson & Johnson Inc., Diamond Bar, CA, United States) or Cool Flex (St. Jude Medical Inc., Minnetonka, MN, United States); 30–35 W, 45°C] was used to deliver radiofrequency energy for ablation under 3D electroanatomical mapping [NavX (St Jude Medical, Minnetonka, MN, United States) or CARTO3 (Johnson & Johnson Inc.)] merged with 3D spiral computed tomography (CT). LA electrogram voltage maps were generated during high right atrial pacing at 500 ms before circumferential pulmonary vein (PV) isolation. However, in minority of patients with recurrent AF at the beginning of the procedure, we acquired voltage maps during sinus rhythm after completion of PV isolation. We obtained the peak-to-peak amplitude of contact bipolar electrograms from 350 to 500 points on the LA endocardium, and the mean LA electrogram voltage was calculated. If frequently recurring AF persisted after three attempts at cardioversion, no further efforts were made to generate an LA voltage map.

All patients initially underwent a circumferential PV isolation. For patients with persistent AF, roof line, posterior inferior line, anterior line, cavotricuspid isthmus line, superior vena cava to septal line, or complex fractionated atrial electrogram-guided ablation were added at the operator’s discretion. The procedure was considered complete when there was no immediate recurrence of AF after cardioversion with an isoproterenol infusion (5–10 μg/min; target heart rate, 120 bpm). In the case of mappable AF triggers or premature atrial beats, non-PV foci were mapped and ablated as much as possible. Systemic anticoagulation was achieved with intravenous heparin while maintaining an activated clotting time of 350–400 s during the procedure.

### Measurement of the LA Pressure, LA Wall Thickness, and LAW-Stress_[*measured*]_

During the AFCA procedure, LA pressure was measured during sinus rhythm and AF immediately after *trans*-septal puncture as described in previous studies (Park et al., 2014, 2019). If the initial rhythm was AF, we measured LA pressure during sinus rhythm after terminating AF by internal cardioversion, followed by at least 3 min waiting period to allow for recovery from atrial stunning from the cardioversion. We excluded those patients in whom LA pressure during sinus rhythm could not be measured due to frequent re-initiations of AF after electrical cardioversion.

We developed a customized software (AMBER, Laonmed Inc., Seoul, South Korea) that measured the LA wall thickness by applying Laplace’s equation in the cardiac CT images (Kwon O. S. et al., 2020; Lee et al., 2021). The number of rows and columns of CT image pixels were 512, and the number of slices was approximately 320 at the *z*-axis. The spatial resolutions of the CT images were within 0.3–0.55 mm for the *x*- and *y*-axis, and the slice thickness of the *z*-axis was 0.5 mm (no overlaps and gaps). The spatial resolution of CT was set to the normalized vector in 3D Euclidean space. The methods and principles of the customized software (AMBER) were previously described in detail, and the results have been well validated with a 3D printed phantom and in 120 patients (Kwon O. S. et al., 2020; Lee et al., 2021). In brief, the endocardium of the LA was semi-automatically divided on the cardiac CT by using the edge detector. Then, the LA wall was extracted with an overlapped area by morphology operations after separation from other tissues using the multi-Otsu threshold algorithm in the histogram of the Hounsfield units. The LA wall thickness was calculated as a numerical streamline connecting the endocardium and epicardium using the Euler method after solving the vector field with Laplace’s equation, the partial differential equation in the 3D space. Then, the mean LA wall thickness was used as a parameter to calculate the LAW-stress.

LAW-stress_[*measured*]_ (dyne/cm^2^) was calculated using the Law of Laplace [σ = (P × r)/2 h (σ, wall stress; P, pressure; r, radius; h, wall thickness)] (Falsetti et al., 1970; Wang et al., 2011). Peak LA pressure during sinus rhythm was directly measured during AF procedure and LA radius was defined as half of the LA anterior–posterior (AP) diameter by transthoracic echocardiography. Therefore, LAW-stress_[*measured*]_ was calculated using the following equation: LAW-stress_[*measured*]_ = (peak LA pressure × LA AP diameter)/(4 × LA wall thickness). LAW-stress was expressed as dyn/cm^2^ (1 mmHg = 1333 dyn/cm^2^).

### AI Model Implementation

We developed a convolutional neural network-based model to classify the risk of LAW-stress_[*measured*]_, as shown in Supplementary Figure 1. The input dimension (8 × 1) was composed of eight non-invasive clinical features [non-paroxysmal type of AF, hypertension, diabetes mellitus, vascular disease, heart failure, left ventricular ejection fraction (LVEF), and the ratio of the peak mitral flow velocity of the early rapid filling to the early diastolic velocity of the mitral annulus (E/Em)], and pre-normalization was performed. The population was randomly divided in a 7:1:2 ratio (training set:validation set:test set). The test and validation sets were scaled with the normalization coefficient (e.g., minimum and maximum) for the training set. Since clinically significant variables were selected as the input variables, only the convolution kernel was considered to avoid the clinical features from being discarded by the pooling operation. The network stream was designed with a typical structure after a convolution filter (3 × 1) to connect with batch normalization and dropout layers and fully connected (FC) layers. The normalized input was performed using batch normalization to reflect the mean and variance of the mini-batch after eight convolution filter operations. The activation function adopted Leaky Rectified Linear Unit (ReLU) to consider the gradient vanishing, and the tensor was serialized (flatten) and then connected with the FC layer. The FC layer consisted of batch normalization, ReLU activation function, and dropout layer, and was recursively connected. The FC layer was a multi-layer perceptron of a two-layer structure, consisting of 16 neurons in the first layer and four neurons in the second layer. The number of convolution filters and number of nodes in the FC layer were selected using a manual search. The output layer used a sigmoid function, and the predicted value ranged from 0 to 1. The whole sample was randomly shuffled, and hyperparameters were conducted by a Bayesian optimizer. The dropout rate was 0.2 and the batch size was 35, and it consisted of stratified sampling to keep the balance between the two classes. The neural network model training was performed with supervised learning using Adam Optimizer (Kingma and Ba, 2014), a backpropagation algorithm, to minimize logit loss calculated by the sigmoid cross-entropy function. The initial learning rate started at 9.36 × 10 ^–3^ and was performed with the cosine annealing methods of a cycle of 20 epochs, and the condition for early stopping was when the logit loss of the validation set stopped improving for 10 epochs. We implemented the software in a developmental environment using Python (ver. 3.5) and TensorFlow (ver. 1.14.0) backend.

### AI Prediction for LAW-Stress

We conducted a quartile analysis for LAW-stress_[*measured*]_ and attempted to detect the highest quartile (Q4) LAW-stress_[*measured*]_ using non-invasive parameters alone. The conventional logistic regression model for the Q4-LAW-stress_[*measured*]_ was derived using a traditional statistical method. Among the variables that had statistically significant associations with LAW-stress_[*measured*]_ in the univariate linear regression analysis, we selected the non-invasive parameters to predict Q4-LAW-stress using AI (Q4-LAW-stress_[AI]_). In the randomly selected training set, five iterations were performed to identify the consistency and robustness of the AI results. Among them, the best-performing model was selected to investigate the association between LAW-stress_[AI]_ and rhythm outcome after AFCA in both cohort 1 and cohort 2. A summary of the study design is shown in Figure 1.

### Post-ablation Management and Follow-Up

All patients visited the scheduled outpatient clinic at 1, 3, 6, and 12 months after the AFCA and every 6 months thereafter or whenever symptoms occurred. All patients underwent electrocardiography at every visit, as well as 24-h Holter recording at 3 and 6 months, then every 6 months for 2 years, annually for 2–5 years, and then biannually after 5 years, following the modified 2012 HRS/EHRA/ECAS expert consensus statement guidelines (Calkins et al., 2012). Whenever patients reported palpitations, Holter monitor or event monitor recordings were obtained and evaluated to check for recurrence of arrhythmias. AF/atrial tachycardia (AT) recurrence was defined as any episode of AF or AT lasting for at least 30 s. Any electrocardiographic documentation of AF/AT recurrence 3 months after the blanking period was diagnosed as a clinical recurrence.

### Statistical Analysis

Continuous variables were expressed as the mean ± standard deviation for normally distributed variables and as the median with the interquartile range for non-normally distributed variables, and compared using the Student’s *t*-test and Wilcoxon rank-sum test, respectively. Categorical variables were reported as counts (percentages) and were compared using the chi-square or Fisher’s exact test. Three or more groups were compared using one-way analysis of variance, and a Bonferroni method was used to account for multiple comparisons between groups. A linear regression analysis was used to investigate the variables related to the LAW-stress_[*measured*]_. The Kaplan–Meier analysis with log-rank test was used to analyze the probability of freedom from AF/AT recurrences after AFCA. A Cox regression analysis was used to identify predictors of AF/AT recurrence after AFCA, and to estimate the hazard ratios (HRs), 95% confidence intervals (CIs), and *p*-values. The variables selected for the multivariate analysis were those with a *p*-value < 0.05 on univariate analysis. Area under the receiver operating characteristic curve (AUC) was used to investigate the predictive power of the AI model and conventional logistic regression model, and clinical outcomes were investigated with Kaplan–Meier analysis. Statistical Package for the Social Sciences version 25.0 for Windows (IBM Corporation, Armonk, NY, United States) and R software version 3.6.2 (The R Foundation for Statistical Computing, Vienna, Austria) were used for data analysis.

## Results

### LAW-Stress_[*measured*]_ Associated Factors

A total of 2223 patients were included in cohort 1 [72.8% male, 59.0 (52.0–67.0) years old, 71.3% with paroxysmal AF (PAF), Table 1] and 658 patients were included in cohort 2 [79.2% male, 57.0 (50.0–65.0) years old, 59.7% with PAF, Supplementary Table 1]. Compared to cohort 1, the patient population included in cohort 2 were younger [59.0 (52.0–67.0) vs. 57.0 (50.0–65.0) years old, *p* < 0.001], had higher proportion of male (72.8% vs. 79.2%, *p* = 0.001), non-paroxysmal AF (28.7% vs. 40.3%, *p* < 0.001), and lower proportion of hypertension (47.1% vs.37.1%, *p* < 0.001) and diabetes (14.9% vs. 8.5%, *p* < 0.001). We obtained LAW-stress based on LA wall thickness, peak LA pressure, and LA diameter in cohort 1; however, the data on LA wall thickness and invasive LA pressure were not available in cohort 2. As shown in Table 1, we divided cohort 1 into four groups based on the quartile values of LAW-stress_[*measured*]_. In the higher quartile LAW-stress_[*measured*]_ group, the patients were older (*p* < 0.001), had a higher proportion of non-paroxysmal AF (*p* < 0.001), body mass index (BMI) (*p* < 0.001), and CHA_2_DS_2_-VASc score (*p* < 0.001), and higher prevalence of hypertension (*p* < 0.001), diabetes mellitus (*p* < 0.001), history of stroke or transient ischemic attack (*p* = 0.041), vascular disease (*p* < 0.001), or heart failure (*p* < 0.001). The LA volume index (*p* < 0.001), LVEF (*p* = 0.028), and E/Em (*p* < 0.001) were higher, and mean LA voltage (*p* < 0.001) was lower in the higher quartile LAW-stress_[*measured*]_ group (Table 1). Procedure-related factors were compared according to the quartiles of LAW-stress_[*measured*]_ in Table 2.

**TABLE 1:** Baseline characteristics according to the quartile value of LA wall stress in cohort 1.

	Overall (*n* = 2223)	Q1 (<97.4 × 10^3^ dyn/cm^2^) (*n* = 556)	Q2 (97.4 to 139.8 × 10^3^ dyn/cm^2^) (*n* = 556)	Q3 (139.8 to 197.9 × 10^3^ dyn/cm^2^) (*n* = 555)	Q4 (≥197.9 × 10^3^ dyn/cm^2^) (*n* = 556)	*P*
Paroxysmal AF, *n* (%)	1576 (71.3)	445 (80.8)^a^	414 (75.1)	406 (73.3)	311 (56.1)^b^	<0.001
Age (years)	59.0 (52.0–67.0)	59.0 (50.0–65.0)^a^	58.0 (50.0–66.0)^a^	59.0 (52.0–67.0)^a^	62.0 (54.0–68.0)^b^	<0.001
Male sex, *n* (%)	1619 (72.8)	417 (75.0)	405 (72.8)	409 (73.7)	388 (69.8)	0.247
**Comorbidities, *n* (%)**						
Hypertension	1046 (47.1)	234 (42.1)^a^	256 (46.0)	254 (45.8)	302 (54.3)^b^	<0.001
Diabetes mellitus	332 (14.9)	61 (11.0)^a^	75 (13.5)	74 (13.3)	122 (21.9)^b^	<0.001
Stroke/TIA	250 (11.2)	48 (8.6)^a^	60 (10.8)	64 (11.5)	78 (14.0)^b^	0.041
Vascular disease	248 (11.2)	45 (8.1)^a^	56 (10.1)	56 (10.1)	91 (16.4)^b^	<0.001
Heart failure	263 (11.8)	55 (9.9)	65 (11.7)	50 (9.0)^a^	93 (16.7)^b^	<0.001
Body mass index (kg/m^2^)	24.7 (23.0–26.7)	24.2 (22.6–25.9)^a^	24.6 (22.8–26.8)^b^	24.8 (23.3–26.9)^b,c^	25.2 (23.4–27.4)^c^	<0.001
CHA_2_DS_2_-VASc score	1.0 (1.0–3.0)	1.0 (0–2.0)^a^	1.0 (0–2.0)^a^	1.0 (0–3.0)^a^	2.0 (1.0–3.0)^b^	<0.001
**Echocardiographic parameters**						
LA dimension (mm)	41.0 (37.0–45.0)	38.0 (34.0–41.0)^a^	40.0 (36.0–44.0)^b^	41.0 (38.0–45.0)^c^	45.0 (41.0–49.0)^d^	<0.001
LA volume index (ml/m^2^)	34.8 (28.0–43.5)	30.3 (25.1–36.9)^a^	33.6 (27.0–40.6)^b^	35.5 (28.8–43.7)^c^	41.2 (33.6–52.3)^d^	<0.001
LV ejection fraction (%)	64.0 (59.0–68.0)	64.0 (60.0–69.0)^a^	64.0 (59.0–69.0)	64.0 (60.0–69.0)^a^	63.0 (58.0–68.0)^b^	0.028
E/Em	9.0 (7.2–12.0)	8.3 (7.0–11.0)^a^	9.0 (7.0–11.0)^a,b^	9.0 (7.9–12.0)^b^	10.3 (8.0–14.0)^c^	<0.001
Mean LA wall thickness (mm)	1.95 (1.75–2.15)	2.06 (1.89–2.27)^a^	2.01 (1.82–2.20)^b^	1.91 (1.73–2.08)^c^	1.80 (1.57–2.01)^d^	<0.001
Mean LA voltage (mV)	1.33 (0.84–1.83)	1.52 (1.06–2.02)^a^	1.43 (0.90–1.91)^a^	1.33 (0.84–1.82)^b^	1.01 (0.65–1.48)^c^	<0.001

*Values are presented as median (Q1–Q3 quartiles [25th and 75th percentiles]) or number (%).^a–d^There were significant differences between the groups with different alphabets. AF, atrial fibrillation; E/Em, ratio of the peak mitral flow velocity of the early rapid filling to the early diastolic velocity of the mitral annulus; LA, left atrial; TIA, transient ischemic attack.*

**TABLE 2:** Procedural characteristics and clinical rhythm outcomes according to the quartile value of LA wall stress in cohort 1.

	Overall (*n* = 2223)	Q1 (<97.4 × 10^3^ dyn/cm^2^) (*n* = 556)	Q2 (97.4 to 139.8 × 10^3^ dyn/cm^2^) (*n* = 556)	Q3 (139.8 to 197.9 × 10^3^ dyn/cm^2^) (*n* = 555)	Q4 (≥197.9 × 10^3^ dyn/cm^2^) (*n* = 556)	*P*
Procedure time (min)	170.0 (138.0–205.0)	159.0 (130.0–185.5)^a^	165.0 (137.0–197.5)^b^	173.0 (140.0–205.0)^b^	188.0 (151.0–228.0)^c^	<0.001
Ablation time (min)	72.7 (53.5–92.0)	66.3 (47.8–80.7)^a^	67.0 (52.6–87.1)^a^	73.8 (54.8–92.3)^b^	83.3 (59.2–105.3)^b^	<0.001
**Ablation lesion, *n* (%)**						
CPVI	2223 (100.0)	556 (100.0)	556 (100.0)	555 (100.0)	556 (100.0)	
SVC-right septal line	1430 (64.4)	339 (61.0)	367 (66.2)	351 (63.2)	373 (67.2)	0.116
Extra PV LA ablation	618 (27.9)	109 (19.7)^a^	105 (19.0)^a^	155 (28.2)	249 (44.8)^b^	<0.001
Extra PV foci, *n* (%)	179 (12.0)	44 (11.7)	37 (9.7)	48 (13.0)	50 (13.5)	0.375
Complications, *n* (%)	80 (3.6)	24 (4.3)	18 (3.2)	21 (3.8)	17 (3.1)	0.661
**Post-ABL medication, *n* (%)**						
ACEi or ARB	777 (35.0)	164 (29.6)^a^	198 (35.6)	189 (34.1)	226 (40.6)^b^	0.002
Beta blocker	838 (37.7)	176 (31.8)^a^	210 (37.8)	207 (37.3)	245 (44.1)^b^	<0.001
Statin	756 (34.0)	169 (30.5)^a^	185 (33.3)^a^	180 (32.4)^a^	222 (39.9)^b^	0.006
Follow-up duration (months)	26.0 (12.0–52.0)	23.0 (11.5–52.5)	26.0 (13.0–52.0)	30.0 (14.0–55.0)	25.0 (11.0–49.0)	0.087
Early recurrence, *n* (%)	666 (30.1)	159 (28.6)^a^	152 (27.4)^a^	160 (29.1)^a^	195 (35.5)^b^	0.015
Clinical recurrence, *n* (%)	821 (37.2)	181 (32.6)^a^	196 (35.3)	190 (34.6)	254 (46.3)^b^	<0.001
Recurrence as paroxysmal type, *n* (% in recur/% in overall)	610 (74.3/27.4)	131 (72.4/23.6)	149 (76.0/26.8)	149 (78.4/26.8)	181 (71.3/32.6)	0.311
AT recurrence, *n* (% in recur/% in overall)	211 (25.7/9.9)	50 (27.6/9.0)	47 (24.0/8.5)	41 (21.6/7.4)	73 (28.7/13.1)	0.311
Cardioversion, *n* (% in recur/% in overall)	301 (36.7/13.5)	45 (24.9/8.1)	66 (33.7/11.9)	74 (38.9/13.5)	116 (45.7/21.1)	<0.001
Recur within 12 months	365 (16.5)	92 (16.5)	84 (15.1)	77 (14.0)^a^	112 (20.4)^b^	0.026
Recur after 12 months	456 (20.6)	89 (16.0)^a^	112 (20.2)	113 (20.6)	142 (25.9)^b^	0.001
Repeat AF ablation, *n* (%)	153 (6.9)	16 (2.9)^a^	30 (5.4)	40 (7.2)	41 (7.4)^b^	0.004
No PV reconnections	33 (21.6)	2 (12.5)	8 (26.7)	11 (27.5)	8 (19.5)	0.032
Extra PV foci during repeat AF ablation	29 (30.5)	1 (14.3)	10 (45.5)	6 (25.0)	9 (27.3)	0.295
Multiple procedure success, overall	105 (64.7)	13 (78.6)	22 (71.4)	26 (63.2)	25 (54.3)	0.332

*Values are presented as median (Q1–Q3 quartiles [25th and 75th percentiles]) or number (%).^a–c^There were significant differences between the groups with different alphabets. ACEi, angiotensin-converting enzyme inhibitor; AF, atrial fibrillation; ARB, angiotensin II receptor blocker; AT, atrial tachycardia; CPVI, circumferential pulmonary vein isolation; LA, left atrial; PV, pulmonary vein; SVC, superior vena cava.*

In the multivariate linear regression analysis, LAW-stress_[*measured*]_ was independently associated with non-paroxysmal AF [β = 31.08 (21.77–40.39), *p* < 0.001], BMI [β = 2.91 (1.53–4.29), *p* < 0.001], diabetes [β = 15.36 (3.35–27.38), *p* = 0.012], vascular disease [β = 22.27 (8.40–36.14), *p* = 0.002], E/Em [β = 4.95 (3.87–6.02), *p* < 0.001], and mean LA voltage [β = −22.24 (−27.96 to −16.52), *p* < 0.001, Table 3].

**TABLE 3:** Linear regression analysis for the clinical variables predictive of LA wall stress (10^3^ dyn/cm^2^) in cohort 1.

	Univariate		Multivariate	
	β (95% CI)	*P*	β (95% CI)	*P*
Non-paroxysmal AF	40.93 (32.52–49.34)	<0.001	31.08 (21.77–40.39)	<0.001
Age	0.83 (0.48–1.18)	<0.001	0.08 (−0.35 to 0.52)	0.713
Male sex	−10.33 (−19.02 to −1.65)	0.020	7.81 (−2.64 to 18.26)	0.143
Hypertension	16.62 (8.90–24.34)	<0.001	3.03 (−6.02 to 12.08)	0.511
Diabetes mellitus	30.17 (19.39–40.94)	<0.001	15.36 (3.35 to 27.38)	0.012
Stroke/TIA	19.16 (6.94–31.37)	0.002	3.00 (−10.6 to 16.59)	0.665
Vascular disease	31.62 (19.41–43.83)	<0.001	22.27 (8.4–36.14)	0.002
Heart failure	34.14 (22.25–46.02)	<.001	−4.79 (−19.99 to 10.40)	0.536
Body mass index	3.71 (2.49–4.92)	<0.001	2.91 (1.53–4.29)	<0.001
Body surface area	14.85 (−5.45 to 35.15)	0.151		
LV ejection fraction	−1.09 (−1.55 to −0.63)	<0.001	−0.07 (−0.66 to 0.51)	0.803
E/Em	5.83 (4.98–6.69)	<0.001	4.95 (3.87–6.02)	<0.001
LVEDD	2.07 (1.20–2.94)	<0.001	0.52 (−0.54 to 1.58)	0.337
Mean LA voltage	−29.36 (−34.94 to −23.78)	<0.001	−22.24 (−27.96 to −16.52)	<0.001

*AF, atrial fibrillation; CI, confidence interval; E/Em, ratio of the peak mitral flow velocity of the early rapid filling to the early diastolic velocity of the mitral annulus; LA, left atrial; TIA, transient ischemic attack; LV, left ventricular; LVEDD, left ventricular end diastolic dimension.*

### LAW-Stress Prediction by the AI Model

To predict Q4-LAW-stress_[*measured*]_, which is a complex parameter requiring an invasive measurement of LA pressure and LA wall thickness for the calculation, we tested the AI prediction accuracy by using common non-invasive clinical variables. Among the variables that were associated with LAW-stress_[*measured*]_ in the univariate linear regression analysis (Table 3), non-paroxysmal type of AF, age, presence of hypertension, diabetes, vascular disease, and heart failure, LVEF, and E/Em were selected for the machine learning algorithm to predict the Q4 of LAW-stress_[*measured*]_ in cohort 1. We excluded the invasive parameter, mean LA voltage, because cohort 2 did not have this invasive variable.

**FIGURE 2:**
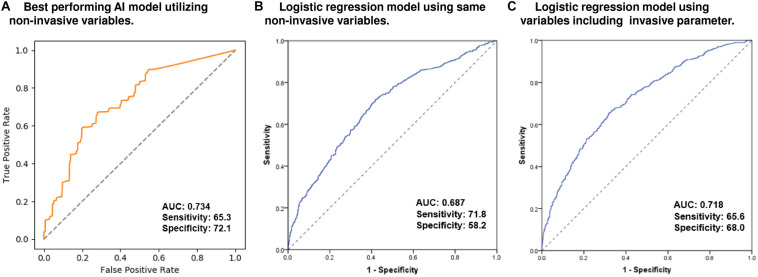
Predictive performance for Q4-LAW-stress_[*measured*]_ in the AI model and conventional logistic regression models. ROC curve of the best-performing AI model **(A)** utilizing non-invasive parameters. ROC curve of the conventional logistic regression model utilizing the same non-invasive parameters **(B)** and utilizing variables including invasive parameters **(C)**. AI, artificial intelligence; AUC, area under the receiver operating characteristic curve; Q4-LAW-stress, highest quartile value of left atrial wall stress; ROC, receiver operating characteristic.

The training time required for the model was approximately 26 min to learn the eight variables from 2223 subjects and the time required to predict the Q4-LAW-stress was approximately 1.8 min. The training, validation, and test sets consisted of randomly selected samples, and all tests were repeated five times. Supplementary Table 2 shows the mean performance results for the Q4-LAW-stress_[*measured*]_ predictions in the training, validation, and test sets. To determine the Q4-LAW-stress_[__AI__]_ in the independent cohort 2, we applied the best-performing model from cohort 1 (AUC 0.734, sensitivity 65.3, specificity 72.1, Gini 0.470, log-loss 0.655, and mean squared error 0.256, Figure 2A). Utilizing the same non-invasive variables, the conventional statistical logistic regression model predicted Q4-LAW-stress_[*measured*]_ with an AUC value of 0.687 (sensitivity 71.8, specificity 58.2, Figure 2B). When we added the invasive parameter, mean LA voltage, the conventional statistical model predicted Q4-LAW-stress_[*measured*]_ with an AUC value of 0.718 (sensitivity 65.6, specificity 68.0, Figure 2C).

### LAW-Stress and the Rhythm Outcome After AFCA

**FIGURE 3:**
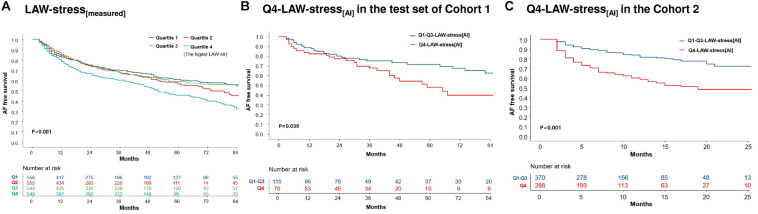
Kaplan–Meier analysis of clinical recurrence of AF after AFCA according to LAW-stress. AF-free survival according to the quartile LAW-stress_[*measured*]_ in cohort 1 **(A)**. AF-free survival according to AI-estimated Q4-LAW-stress_[AI]_ in cohort 1 **(B)** and cohort 2 **(C)**. AF, atrial fibrillation; AFCA, atrial fibrillation catheter ablation; AI, artificial intelligence; LAW-stress, left atrial wall stress; Q4-LAW-stress, the highest quartile value of left atrial wall stress.

During 26.0 (12.0–52.0) months of follow-up, AF/AT recurrence was significantly higher in the Q4-LAW-stress_[*measured*]_ group (log-rank *p* < 0.001, Figure 3A) and Q4-LAW-stress_[AI]_ group (log-rank *p* = 0.039, Figure 3B) in cohort 1. In the multivariate Cox regression analysis for clinical recurrence in cohort 1, LAW-stress_[*measured*]_ [HR 2.43 (1.21–4.90), *p* = 0.013], non-paroxysmal AF [HR 1.61 (1.39–1.87), *p* < 0.001], and female sex [HR 1.20 (1.03–1.40), *p* = 0.023] were independently associated with clinical recurrence of AF after AFCA (Table 4). In cohort 2, the Q4-LAW-stress_[AI]_ group consistently had worse rhythm outcome (log-rank *p* < 0.001, Figure 3C). In the multivariate Cox regression analysis in cohort 2, Q4-LAW-stress_[AI]_ [HR 2.19 (1.54–3.11), *p* < 0.001], age [HR 0.97 (0.95–0.98), *p* < 0.001], AF duration [HR 1.03 (1.00–1.06), *p* = 0.026], and LA dimension [HR 1.05 (1.02–1.08), *p* = 0.001] were independently associated with clinical recurrence of AF after AFCA (Supplementary Table 3).

**TABLE 4:** Cox regression analysis for clinical recurrence of AF in cohort 1.

	Univariate		Multivariate	
	HR (95% CI)	*P*	HR (95% CI)	*P*
Non-paroxysmal AF	1.68 (1.48–1.90)	<0.001	1.61 (1.39–1.87)	<0.001
Female sex	1.13 (0.99–1.29)	0.074	1.20 (1.03–1.40)	0.023
Age	1.00 (1.00–1.01)	0.323	1.00 (0.99–1.01)	0.663
Hypertension	1.09 (0.96–1.23)	0.178		
Diabetes mellitus	1.10 (0.93–1.30)	0.256		
Stroke/TIA	1.16 (0.96–1.39)	0.123		
Heart failure	1.24 (1.03–1.50)	0.023	1.01 (0.80–1.28)	0.916
Vascular disease	1.05 (0.87–1.26)	0.626		
Body mass index	1.01 (0.99–1.03)	0.362		
LV ejection fraction	0.99 (0.99–1.00)	0.021	1.00 (0.99–1.01)	0.332
E/Em > 15	1.15 (0.95–1.39)	0.145		
LVEDD	1.01 (1.00–1.03)	0.064		
LA wall stress (per dyn/cm^2^)	4.36 (2.27–8.38)	<0.001	2.43 (1.21–4.90)	0.013

*AF, atrial fibrillation; CI, confidence interval; E/Em, ratio of the peak mitral flow velocity of the early rapid filling to the early diastolic velocity of the mitral annulus; eGFR, estimated glomerular filtration rate; HR, hazard ratio; LA, left atrial; LV, left ventricular; LVEDD, left ventricular end diastolic dimension; TIA, transient ischemic attack.*

**FIGURE 4:**
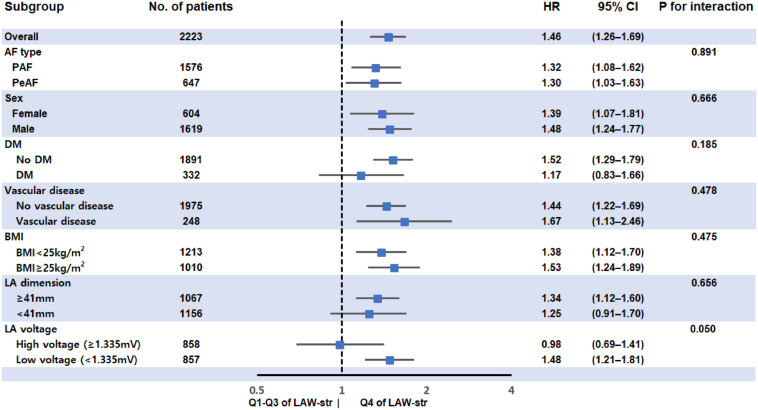
Subgroup analysis for rhythm outcome after AFCA according to LAW-stress_[*measured*]_. LAW-stress_[*measured*]_ was independently associated with post-AFCA recurrence, regardless of AF type, sex, BMI, or associated vascular disease. BMI values were divided into two groups using a BMI of 25 kg/m^2^, which is the cutoff value for being overweight. The LA dimension and LA voltage were divided by the median values. AF, atrial fibrillation; AFCA, atrial fibrillation catheter ablation; BMI, body mass index; CI, confidence interval; DM, diabetes mellitus; HR, hazard ratio; LAW-stress, left atrial wall stress; PAF, paroxysmal atrial fibrillation; PeAF, persistent atrial fibrillation.

In the subgroup analyses, LAW-stress_[*measured*]_ was independently associated with post-AFCA recurrence, regardless of AF type, sex, BMI, or presence of vascular disease (Figure 4).

## Discussion

### Main Findings

In the present study, we calculated LAW-stress_[*measured*]_ using LA pressure, dimension, and wall thickness, and evaluated its prognostic value in patients with AF after catheter ablation. LAW-stress_[*measured*]_ was independently related to non-paroxysmal AF, diabetes, vascular disease, BMI, E/Em, and low mean LA voltage. We also estimated this complicated parameter (LAW-stress_[AI]_) based on non-invasive common clinical variables using AI. AF recurrence was significantly higher in both the higher LAW-stress_[*measured*]_ and LAW-stress_[AI]_ groups. The high LAW-stress_[AI]_ group consistently had worse rhythm outcomes after AFCA in the independent cohort. AI was able to predict this complex but useful prognostic parameter using non-invasive variables with moderate accuracy.

### Role of LAW-Stress in the Mechanism of AF

Chronic atrial stretch causes atrial dilatation and heterogeneous changes in atrial cellular structures (Takeuchi et al., 2006). Although the association between cardiac wall tension and ventricular remodeling is well known, direct comparisons of LAW-stress and atrial remolding have been very limited (Pouleur et al., 1993; Burchfield et al., 2013). This is because the atrial structure is complex, LA pressure measurements require an invasive procedure, and there is no standard for measuring thin atrial wall thickness.

Therefore, we calculated this complex parameter, the LAW-stress_[*measured*]_, by using the direct LA pressure measured during AFCA (Park et al., 2014, 2019) and CT-based mean LA wall thickness measured using customized software (AMBER, Laonmed, South Korea) (Kwon O. S. et al., 2020). LAW-stress is a comprehensive parameter that reflects not only LA size but also LA hemodynamic status and innate patient characteristics, such as regional LA wall thickness. In this study, LAW-stress_[*measured*]_ had significant associations with chronic atrial remodeling and left ventricular diastolic dysfunction, such as persistent AF, low LA voltage, high BMI, and E/Em, which have been reported to be related to poor rhythm outcomes (Park et al., 2009; Kim I. S. et al., 2015; Baek et al., 2016). A recent study also indicated that LA compliance at baseline was associated with LA reverse remodeling after AFCA (Tops et al., 2011).

### LAW-Stress as a Predictive Marker of AF Recurrence

Although AFCA is an effective but invasive rhythm control strategy, the long-term recurrence rate is substantial, especially in patients with longstanding persistent AF. LA size reflects the degree of remodeling or progression of AF and is known to be related to the risk of recurrence after AFCA, but some studies have reported inconsistent results (Zhuang et al., 2012; Njoku et al., 2018). This is because LA size, which is a simple anatomical index, is affected by various pathophysiological conditions, such as electrical and structural remodeling, hemodynamic conditions, or underlying pathophysiology. For example, successful rhythm control by AFCA reduces LA size remarkably, while LA hemodynamic unloading by mitral valve surgery also contributes to successful AF rhythm control (John et al., 2010; Zhuang et al., 2012; Kim et al., 2019). On the other hand, LAW-stress is a complex and comprehensive prognostic factor with a higher specificity for rhythm prognosis in consideration of innate LA wall thickness, histopathological changes, and hemodynamic burden.

### Role of AI in the Prediction of High LAW-Stress

Recently, AI has been applied to cardiovascular medicine in various ways (Choi et al., 2017; Krittanawong et al., 2017). AI has been tested for diagnosing cardiac diseases, and its high prognostic predictive power in cardiac images and electrocardiograms has already been verified (Arnar et al., 2006; Betancur et al., 2018). We saw another potential for AI in that it could be useful for predicting invasive and complex parameters with a diagnostic and prognostic value by substituting them with non-invasive common variables in this study. Using AI, we predicted LAW-stress_[AI]_, which, despite having clinical value, was otherwise complex, difficult to calculate, and included invasive parameters (Falsetti et al., 1970; Wang et al., 2011), and validated its prognostic value in an independent cohort. Further prospective studies with a large sample size are warranted.

### Study Limitations

There were several limitations to this study. First, as the left atrium is not an exact sphere, the Law of Laplace may not be definitely suited for LAW-stress. To calculate the global LAW-stress, we assumed that there were no regional differences of wall thickness in the left atrium. Second, although we waited for LA pressure stabilization at least for 3 min in each patient (Park et al., 2014, 2019; Kim T. H. et al., 2015; Pak et al., 2021), the mechanical stunning of the LA after cardioversion may affect the LA pressure. Third, although the results were validated with other datasets, this study was mainly performed using single center data. Therefore, generalization of the results should be considered with circumspection. Fourth, the number of patients may not be sufficient for developing an AI model. To reduce this limitation, we selected training and validation sets five times randomly from our cohort data. Fifth, since the cohort 2 database did not have data on LA pressure and LA wall thickness, validation of the AI model for LAW-stress was performed indirectly by predicting the rhythm outcome of the estimated LAW-stress groups. In addition, due to the different time of enrollment and follow-up duration between the development and independent cohorts, differences in catheter type, ablation lesion set, and AF recurrence between the two cohorts should be considered. However, there were previous AI-related studies analyzing results with data that have a time discrepancy of enrollment in training cohort and validation cohort (Feeny et al., 2020; Firouznia et al., 2021).

## Conclusion

A higher LAW-stress was associated with poorer rhythm outcomes after AFCA, and AI was able to predict this complex but useful prognostic parameter using non-invasive parameters with moderate accuracy.

## Data Availability Statement

The original contributions presented in the study are included in the article/Supplementary Material, further inquiries can be directed to the corresponding author/s.

## Ethics Statement

The studies involving human participants were reviewed and approved by Institutional review boards of the Yonsei University Health System and Korea University Cardiovascular Center. The patients/participants provided their written informed consent to participate in this study.

## Author Contributions

J-HL, O-SK, JS, and H-NP conceived and designed the study, performed the statistical analysis, and drafted manuscript. JS, HY, T-HK, J-SU, BJ, M-HL, Y-HK, and H-NP recruited study subjects. O-SK, JL, and H-JH programmed artificial intelligence and performed technical support. All authors have read and approved the final manuscript.

## Conflict of Interest

The authors declare that the research was conducted in the absence of any commercial or financial relationships that could be construed as a potential conflict of interest.
