# Supplementary material for: Left Atrial Wall Stress and the Long-Term Outcome of Catheter Ablation of Atrial Fibrillation: An Artificial Intelligence-Based Prediction of Atrial Wall Stress
Source: Front Physiol. 2021 Jul 2;12:686507. doi: 10.3389/fphys.2021.686507 (PMC8285096; doi:10.3389/fphys.2021.686507)
Supplement: Supplementary file 1 [file Data_Sheet_1.docx]

Supplementary Material

**Supplementary Figure 1. Artificial intelligence architecture.**

**Supplementary Table 1. Baseline characteristics and rhythm outcomes of the patients in Cohort 2.**

**Supplementary Table 2. Mean predictive performance of the AI model for Q4 of LAW-stress_[measured]_ in Cohort 1.**

**Supplementary Table 3. Cox regression analysis for clinical recurrence of AF in Cohort 2.**


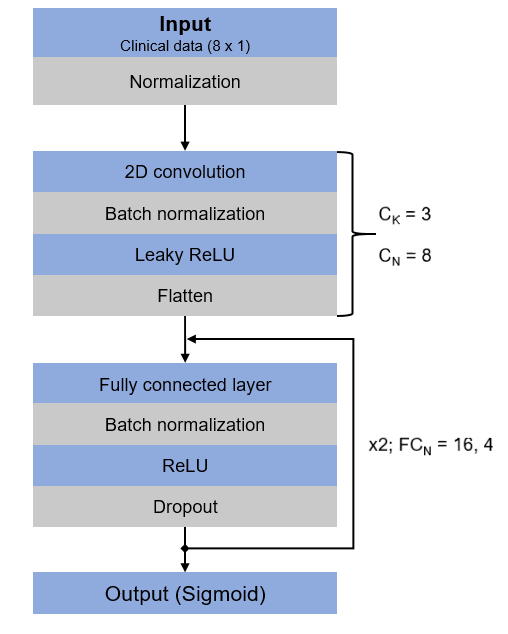


**Supplementary Figure 1. Artificial intelligence architecture.**

C_K_, the size of convolution kernels; C_N_, the number of convolution filters; FC_N_, the number of neurons of fully connected layers.

**Supplementary Table 1. Baseline characteristics and rhythm outcomes of the patients in Cohort 2.**

|  | Cohort 2 (n=658) |
| --- | --- |
| Paroxysmal AF, n (%) | 393 (59.7) |
| Age (years) | 57.0 (50.0–65.0) |
| Male sex, n (%) | 521 (79.2) |
| Comorbidities, n (%) | |
| Hypertension | 244 (37.1) |
| Diabetes mellitus | 56 (8.5) |
| Stroke/TIA | 59 (9.0) |
| Vascular disease | 45 (6.8) |
| Heart failure | 10 (1.5) |
| Body mass index (kg/m^2^) | 24.6 (22.9–26.6) |
| CHA_2_DS_2_-VASc score | 1.0 (0.0–2.0) |
| Echocardiographic parameters | |
| LA dimension (mm) | 41.1 (37.7–44.9) |
| LV ejection fraction (%) | 57.5 (54.5–57.5) |
| E/Em | 8.4 (6.7–10.5) |
| Clinical recurrence, n (%) | 174 (26.4) |

*Values are presented as median (Q1–Q3 quartiles [25th and 75th percentiles]) or number (%).*

*E/Em, the ratio of the peak mitral flow velocity of the early rapid filling to the early diastolic velocity of the mitral annulus; LA, left atrial; LV, left ventricular; TIA, transient ischemic attack.*

**Supplementary Table 2. Mean predictive performance of the AI model for Q4 of LAW-stress_[measured]_ in Cohort 1.**

| Data set | AUC | Sensitivity | Specificity | PPV | NPV | Gini | Log–loss | MSE | Accuracy |
| --- | --- | --- | --- | --- | --- | --- | --- | --- | --- |
| Training set | 0.727  (0.716 – 0.738) | 0.699  (0.665 – 0.732) | 0.687  (0.664 – 0.71) | 0.419  (0.406 – 0.431) | 0.876  (0.865 – 0.888) | 0.454  (0.433 – 0.476) | 0.612  (0.572 – 0.652) | 0.215  (0.198 – 0.231) | 0.690  (0.678 – 0.702) |
| Validation set | 0.688  (0.658 – 0.718) | 0.636  (0.594 – 0.678) | 0.695  (0.664 – 0.726) | 0.456  (0.424 – 0.488) | 0.823  (0.781 – 0.865) | 0.376  (0.317 – 0.435) | 0.656  (0.634 – 0.678) | 0.244  (0.218 – 0.27) | 0.677  (0.651 – 0.703) |
| Test set | 0.694  (0.672 – 0.715) | 0.655  (0.606 – 0.705) | 0.660  (0.612 – 0.707) | 0.412  (0.379 – 0.444) | 0.842  (0.821 – 0.863) | 0.387  (0.344 – 0.43) | 0.645  (0.600 – 0.691) | 0.239  (0.215 – 0.263) | 0.657  (0.631 – 0.683) |

*Values are presented as average (95% confidence interval).*

*AUC, area under the curve; CI, confidence interval; LAW-stress, left atrial wall stress; MSE, mean squared error; NPV, negative predictive value; PPV, positive predictive value.*

**Supplementary Table 3. Cox regression analysis for clinical recurrence of AF in Cohort 2.**

|  | Univariate | | Multivariate | |
| --- | --- | --- | --- | --- |
|  | HR (95% CI) | P | HR (95% CI) | P |
| Age | 0.98 (0.96–0.99) | 0.002 | 0.97 (0.95–0.98) | <0.001 |
| Sex | 1.40 (0.94–2.08) | 0.094 |  |  |
| Body mass index | 1.08 (1.03–1.13) | 0.001 | 1.01 (0.96–1.06) | 0.787 |
| Non-paroxysmal AF^1^ | 3.11 (2.27–4.24) | <0.001 |  |  |
| AF duration | 1.03 (1.00–1.05) | 0.024 | 1.03 (1.00–1.06) | 0.026 |
| Heart failure | 0.42 (0.06–2.98) | 0.383 |  |  |
| Hypertension | 0.98 (0.72–1.33) | 0.875 |  |  |
| Diabetes | 1.43 (0.88–2.33) | 0.154 |  |  |
| Stroke/TIA | 1.33 (0.83–2.11) | 0.236 |  |  |
| Vascular disease | 0.74 (0.37–1.51) | 0.413 |  |  |
| CHA_2_DS_2_VASc score | 0.95 (0.84–1.08) | 0.445 |  |  |
| LV ejection fraction | 0.99 (0.97–1.02) | 0.644 |  |  |
| LA dimension | 1.07 (1.04–1.10) | <0.001 | 1.05 (1.02–1.08) | 0.001 |
| E/Em | 0.96 (0.91–1.01) | 0.104 |  |  |
| Q4-LAW-stress_[AI]_ | 2.78 (2.03–3.81) | <0.001 | 2.19 (1.54–3.11) | <0.001 |

*^1^ Non-paroxysmal AF was not included in multivariate analysis due to multicollinearity with Q4-LAW-stress_[AI]_*

*AF, atrial fibrillation; CI, confidence interval; E/Em, ratio of the peak mitral flow velocity of the early rapid filling to the early diastolic velocity of the mitral annulus; HR, hazard ratio; LA, left atrial; LV, left ventricular; TIA, transient ischemic attack.*
